# Low-Frequency rTMS and Diazepam Exert Synergistic Effects on the Excitability of an SH-SY5Y Model of Epileptiform Activity

**DOI:** 10.3390/biomedicines13081857

**Published:** 2025-07-30

**Authors:** Ioannis Dardalas, Efstratios K. Kosmidis, Roza Lagoudaki, Vasilios K. Kimiskidis, Theodoros Samaras, Theodoros Moysiadis, Dimitrios Kouvelas, Chryssa Pourzitaki

**Affiliations:** 1 Laboratory of Clinical Pharmacology, Aristotle University of Thessaloniki, 54124 Thessaloniki, Greece; 2 Laboratory of Physiology, Department of Medicine, Aristotle University of Thessaloniki, 54124 Thessaloniki, Greece; 3 First Department of Neurology, AHEPA University Hospital, Aristotle University of Thessaloniki, Stilponos Kyriakidi 1, 54636 Thessaloniki, Greece; 4 Faculty of Sciences, School of Physics, Aristotle University, 54124 Thessaloniki, Greece; 5 Department of Physics, University of Malta, 2080 Msida, Malta; 6 Department of Computer Science, School of Sciences and Engineering, University of Nicosia, 2417 Nicosia, Cyprus

**Keywords:** magnetic stimulation, diazepam, in vitro, cell culture, epileptogenesis, antiepileptic, calcium signaling

## Abstract

**Background/Objectives:** Epilepsy is a brain condition that affects millions of people worldwide. Although there are many antiepileptic drugs with different mechanisms of action, many patients still fail to control their agonizing symptoms, a situation that highlights the need for more strategies to address this issue. In this in vitro study, we elucidated and characterized the alterations in intracellular Ca^2+^ levels in cell cultures where diazepam and repetitive transcranial magnetic stimulation were implemented, alone or in combination. **Methods:** Using the differentiated human-derived neuroblastoma cell line SH-SY5Y, we measured the alterations in intracellular Ca^2+^ levels under the impact of either low-frequency repetitive transcranial magnetic stimulation (1 Hz), diazepam (14 μM), or their combination. We used the Ca^2+^-sensitive fluorescent indicator Fluo-4 acetoxymethyl ester for calcium imaging, while neuronal excitation was achieved with 50 mM KCl. **Results:** The highest median fluorescence intensity increase (%ΔF/F = 24.80) was observed in control cell cultures, followed by rTMS cultures (%ΔF/F = 16.96) and diazepam cultures (%ΔF/F = 11.46). The lowest median fluorescence intensity value (%ΔF/F =−0.44) was observed when diazepam was used concomitantly with repetitive transcranial magnetic stimulation. Post hoc analysis assessed pairwise differences, showing statistically significant differentiation between the control group and all other groups. Additionally, statistically significant results were observed between repetitive transcranial magnetic stimulation or diazepam and their combination, but not between them. **Conclusions:** The combination of diazepam and repetitive transcranial magnetic stimulation resulted in the most significant reduction in intracellular Ca^2+^ levels, as indicated by the lowest fluorescence values compared with the control group. Individually, each treatment produced a notable but less pronounced effect. We conclude that both diazepam and low-frequency repetitive transcranial magnetic stimulation can control epileptiform activity in vitro, while their combination is the most effective treatment.

## 1. Introduction

Epilepsy affects approximately 7.60 per 1000 individuals throughout their lives. Its prevalence and incidence show global heterogeneity [1]. The condition is defined by the recurrence of spontaneous seizures [2]. Epilepsy most commonly affects the youngest and oldest age groups, and males show a higher incidence than females [1]. Epileptic patients often experience anxiety, depression, sleep or movement disorders, and migraines, all of which must be considered during their treatment planning [3].

Despite the introduction of new antiepileptic drugs, many individuals may not remain seizure-free, a state of affairs that has not significantly changed over the past twenty years [3]. Some patients continue to experience seizures and do not respond effectively to a single therapy or combined drug therapies, a condition known as drug-resistant epilepsy (DRE) [4].

Diazepam, a benzodiazepine, acts as a GABA agonist and is widely used as a sedative, anxiolytic, sleeping aid, and antiepileptic drug to effectively manage seizures, either alone or as an adjunct therapy to other treatments [5,6,7,8]. Diazepam has been used as an antiepileptic drug in recent decades to control various types of seizures [9]. Its current use includes nasal administration for rapid seizure resolution [10]. Rectal diazepam can also prove beneficial to terminate cluster seizures during emergencies [11].

Repetitive transcranial magnetic stimulation (rTMS) is a non-invasive method of brain stimulation that exerts excitability alterations in the motor cortex. TMS may impact synaptic plasticity and produce long-lasting effects [12]. It has found application in various conditions or diseases, namely, postoperative pain, Parkinson’s disease, multiple sclerosis (MS), Alzheimer’s disease, and epilepsy [13]. The characteristics of the rTMS protocol utilized, namely, the field intensity, frequency, number of pulses, and trains, should be well established to achieve the desired effects. Several different protocols for repetitive transcranial magnetic stimulation (rTMS) have yielded diverse outcomes. Low-frequency rTMS, typically at 1 Hz or below, has an inhibitory effect on brain activity, while high-frequency rTMS, usually above 5 Hz, is considered excitatory, promoting neuronal activation. The differentiation may influence how rTMS is applied in therapeutic protocols [12].

Although rTMS is a well-established therapeutic method with a wide range of applications across multiple neurological and psychiatric conditions, the underlying mechanisms remain unknown. To address this gap, we conducted an in vitro experiment using the human neuroblastoma cell line SH-SY5Y. Cell cultures were differentiated into mature neuronal cultures, characterized by elongated neurites and the expression of neuronal markers, providing a robust model for studying neuronal behavior [14]. Previous research has demonstrated that intracellular Ca^2+^ ion concentration notably increases during epileptiform activity, correlating with neuronal excitability and contributing to seizure activity [15].

Intracellular calcium, a mediator of neuronal excitability and seizure activity, plays an important role in epileptogenesis. Several studies have tried to elucidate their correlation [16]. A study in guinea pig hippocampal slices used two models of epilepsy, implementing either bicuculline or low Mg^2+^, and found that the calcium transients were five times larger than in controls during epileptiform activity. The results indicated that cell somata and basal dendrites may be responsible for the epileptiform spread [17].

Another study used neocortical slices, where epileptiform activity was induced via either low magnesium, bicuculline, or 4-aminopyridine, and found a strong correlation between Ca^2+^ levels and epileptiform activity, confirmed via microfluorimetric imaging. The researchers used the antiepileptic drugs levetiracetam and lamotrigine, and both drugs dose-dependently reduced Ca^2+^ elevation, suggesting anticonvulsant efficacy and augmented neuronal viability [18]. In an in vitro study, a magnesium-free bathing solution induced epileptiform activity in the prefrontal cortex. The researchers proposed that internal calcium stores are responsible for synchronized epileptiform activity in vitro and may contribute to pathological and physiological synchronized cortical activity in vivo [19].

To mimic “seizure-like events” (SLEs) in vitro (thereby partially replicating seizures observed in vivo), researchers use specific methods such as manipulating ion concentrations or employing pharmacological agents that target glutamate or GABA receptors. Changes in ion levels can entail altering the bathing solution to increase potassium levels or decrease calcium and magnesium levels [16].

The aim of our research was to study the combined effects of diazepam and TMS in vitro, using the fluorescent chemical Ca^2+^ indicator Fluo-4 AM, a useful tool for quantifying intracellular Ca^2+^ concentrations under different conditions.

## 2. Materials and Methods

### 2.1. Chemicals and Reagents

This in vitro study was performed using a commercially available cell line and reagents, with no involvement of human subjects or laboratory animals; therefore, neither informed consent nor animal ethics approval was applicable. Diazepam was obtained from Stedon (Athens, Greece). Eagle’s minimum essential medium (EMEM), Dulbecco’s modified Eagle’s medium (DMEM), fetal bovine serum (FBS), and Pluronic F-127 were obtained from Sigma-Aldrich (St. Louis, MO, USA). Neurobasal medium without phenol red, dimethylsulfoxide (DMSO), penicillin–streptomycin, N2 supplement, and trypsin–EDTA (1×) were obtained from Gibco™. Glutamine (100×) (200 mM) was purchased from Biowest. Human brain-derived neurotrophic factor (BDNF) was acquired from Abbkine (Abbkine Scientific Co., Ltd., Wuhan, China). Fluo-4, AM (acetoxymethyl ester), was purchased from Invitrogen (by Thermo Fisher Scientific) (Waltham, MA, USA). A KCl (50 mM) solution was used to promote excitation and dynamically increase the intracellular Ca^2+^ [20].

### 2.2. Cell Culture

The human neuroblastoma cell line SH-SY5Y was purchased from Sigma-Aldrich (St. Louis, MO, USA) and used according to the manufacturer’s guidelines and standardized protocols, consistent with GIVIMP requirements. The cells were grown in a humidified incubator (5% CO_2_; 37 °C) in T25 cell culture flasks in EMEM supplemented with glutamine, penicillin–streptomycin, and FBS through passages until the onset of the differentiation protocol for each batch. The experimental cells were divided into 4 groups: a control group, a diazepam group, an rTMS group, and a diazepam-plus-rTMS group.

### 2.3. Differentiation Protocol

A 6-day two-step protocol proposed by Forster et al. was used, which included two phases with different medium compositions [21]. For the initiation of the differentiation protocol, the cells were harvested and centrifuged at 1500 rpm for 10 min and then directly seeded in the incubator on 35 mm × 10 mm Petri dishes for 24 h. After 24 h, the phase 1 medium was added to the cell cultures, containing 10 µM all-trans retinoic acid, 4 mM L-glutamine, and 1% penicillin–streptomycin in high-glucose (25 mM) DMEM without sodium pyruvate. After 72 h, the phase 2 medium was added to the cell cultures, replacing the phase 1 medium, containing 1% 100× (*v*/*v*) N-2 supplement, 50 ng/mL (*v*/*v*) brain-derived neurotrophic factor (BDNF), 1% penicillin–streptomycin, and 1% L-glutamine in neurobasal medium (NB) without phenol red. Three days after the addition of the phase 2 medium, the cell cultures were ready for calcium imaging.

### 2.4. Calcium Imaging

Fluo-4, AM, belongs to a group of molecules called labeled Ca^2+^ indicators. Importantly, they can present a rise in fluorescence after binding to Ca^2+^. In our experiment, Fluo-4, AM, was solubilized in DMSO (50 μg of Fluo-4, AM, in 0.2 mL of DMSO). The loading buffer for each of the Petri dishes (35 mm × 10 mm) consisted of 1.5 mL DMEM, Fluo-4, AM (5 μM), and 6 μL of Pluronic F-127 (20% in DMSO *w*/*v*). The cells were incubated at 37 °C for 30 min before the loading buffer was removed and replaced by DMEM with FBS (15%) for a second incubation of 15 to 30 min at room temperature [22].

A first calcium imaging protocol was performed. The cells were then treated with a 50 mM KCl solution to induce membrane depolarization while also augmenting the intracellular Ca^2+^, before a second similar calcium imaging protocol was implemented. Neurobasal medium minus phenol red was used during the calcium imaging, which was essential to meet the neuronal requirements.

The acquisition parameters in our calcium imaging recording protocol, which was identical either before or after the addition of KCl, included a 128 × 128 configuration, with a 10 ms frame interval and 512 frames for acquisition for a total duration of 5120 ms for each trial. In total, 10 trials were recorded, with a 10 sec interval between the trials. The microscope field extraction recordings were then processed and analyzed for variations in fluorescence.

In our experiments, the microscope used to optically measure and quantify the Ca^2+^ was a Carl Zeiss Axio Examiner.Z1 (Carl Zeiss Microscopy GmbH, Jena, Thuringia, Germany) with a Zeiss 10×/0.3 water immersion objective lens. A high-speed CMOS camera, NeuroCMOS-SM128, (Redshirt Imaging Inc., Decatur, GA, USA) was connected to the c-mount port of an upright epifluorescence microscope and was used for optical recordings throughout our experiments. The software Neuroplex version 9.9.8. was used to analyze the imaging data throughout our study.

### 2.5. ΔF/F

Ten traces with a Kernel size of 2 pixels, each one falling within a unique different cell, of the microscope field were chosen to measure the intracellular Ca^2+^ alterations after adding KCl. Cells with extreme fluorescence values were not included. The identical traces were tracked after adding KCl. In calcium imaging studies, the amount of fluorescence is based on the Ca^2+^ concentration and, equally, on the amount of the Ca^2+^-sensitive fluorophore inside the cells. Thus, the traces were calculated as %ΔF/F (meaning the fluorescence variation divided by the resting fluorescence intensity value, or the fluorescence before the addition of KCl) in correlation with the intracellular Ca^2+^ concentration to normalize the fluorescence levels [23,24].

### 2.6. Diazepam

We used diazepam at a concentration of 14 μM for pharmacological treatment, which aligns with levels reported in prior studies [8,25,26]. This dose was chosen as it effectively influenced the cells without compromising their viability or altering the culture’s confluency. The cells were treated with diazepam 30 min before conducting calcium imaging.

### 2.7. rTMS

We used the transcranial magnetic stimulation unit MagPro R30 MagVenture (MagVenture A/S, Farum, Copenhagen, Denmark) with a C-B60 figure-8 coil to perform magnetic stimulation on our cells. Our protocol included 2 trains with 100 pulses per train for a total of 200 pulses at a frequency of 1 Hz and 100% amplitude. The coil temperature never exceeded 40 °C during the procedure, which was verified with an infrared thermometer (Kessler Thermometer Corp., West Babylon, NY, USA). The Petri dish was placed at the vertical and horizontal center of the figure-8 coil to achieve the maximum magnetic intensity.

**Figure 1:**
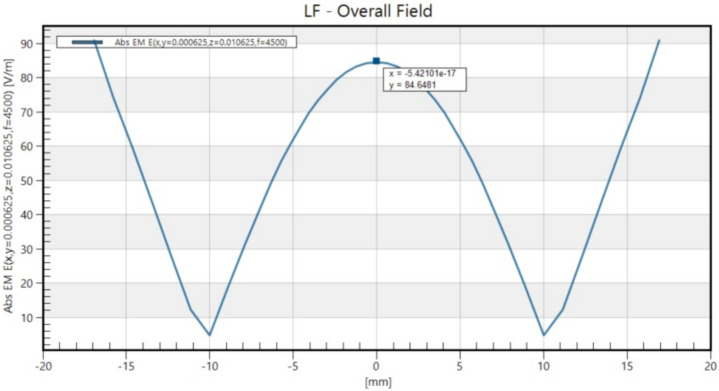
The spatial distribution of the electromagnetic field strength that aligned with the long axis of the figure-8 coil.

The electric field induced within the dish was calculated using Sim4Life (version 7.2, ZMT Zurich MedTech AG, Zurich, Switzerland). A harmonic current with a frequency of 4.5 kHz was assumed, corresponding to the basal frequency of a rectangular pulse with a 222 μs duration. The electrical conductivity used in the simulation was set to 1.5 S/m, as measured in Khire et al.’s study [27]. The resulting maximum magnitude of the magnetic field at the bottom of the culture medium was 0.98 T, whereas the maximum induced electric field inside the culture medium was 90 V/m (Figure 1).

### 2.8. Statistical Analysis

The statistical analysis used standard descriptive statistics to evaluate the percentage change in the fluorescence intensity (% ΔF/F) within the four different categories of treatment (control, TMS, diazepam, and TMS and diazepam). For visualization purposes, the R package ‘ggstatsplot’ (version 0.12.4) was used with a combination of box and violin plots [28]. The non-parametric Kruskal–Wallis test was performed to evaluate the differences in the distributions of % ΔF/F among these four categories. Parametric analysis of variance (ANOVA) was not selected since the normality assumption was not satisfied in all categories. Dunn’s test was used to perform multiple pairwise comparisons, along with the Benjamini–Hochberg procedure to account for multiple comparisons [29]. The level of statistical significance was set at 0.05. The analyses were conducted using the SPSS software (version 22.0) and the R programming language (version 4.4.1).

## 3. Results

In our experiment, we implemented two different differentiation protocols, namely, the 17-day multistep protocol proposed by Shipley et al. and the 6-day two-step protocol proposed by Forster et al. [21,30]. The cells were stable for 14 days following differentiation [21]. We concluded that the optimal differentiation protocol for our experiment was the one proposed by Forster et al. [21], as it resulted in a more favorable cell confluency of 70 to 90%, where confluency reflects the percentage of the culture dish surface covered by adherent cells. This 6-day protocol was also more time-efficient while requiring fewer laboratory resources.

Before initiating our experiments, we performed a pilot study to measure the action of KCl on the differentiated SH-SY5Y cell line treated with Fluo-4, AM. Its effects on fluorescence intensity augmentation were rapid and consistent [16]. After completing the pilot study, we proceeded with repeated experiments comparing cell cultures with no intervention, cell cultures with pharmacological intervention, cell cultures with magnetic stimulation, and cell cultures with both pharmacological intervention and magnetic stimulation.

As described in previous studies, adding 50 mM KCl to the bath solution will increase the Ca^2+^ concentration, consequently increasing the fluorescence intensity [16,20,31]. As expected, a rapid and notable rise in intracellular Ca^2+^ was observed in our study after adding the KCl solution to the media. The rTMS intervention throughout our study was implemented at 1 Hz. All data regarding the pharmacological intervention with diazepam, as displayed in Table 1, were extracted from experiments consistently performed at a concentration of 14 μΜ.

As described in Table 1, the highest observed fluorescence intensity increase (comparison before and after adding KCl) was recorded in the control cell cultures (%ΔF/F = 24.80), which had no pharmacological intervention or magnetic stimulation. The lowest variation in fluorescence intensity was observed in the cell cultures with both pharmacological intervention and magnetic stimulation (rTMS) (%ΔF/F = −0.44). Between these two, the cell cultures with only pharmacological intervention and those with only magnetic stimulation had median %ΔF/F values of 11.46 and 16.96, respectively.

**Figure 2:**
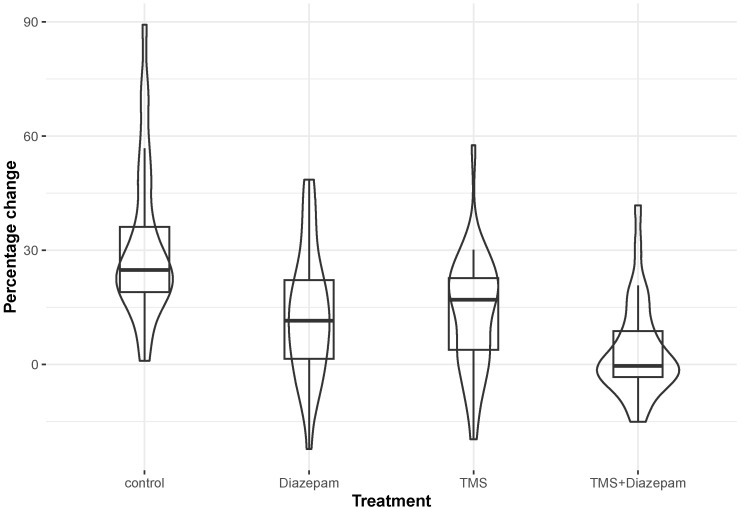
A combination of box and violin plots to display the percentage change in fluorescence within each of the four treatment categories (control, TMS, diazepam, and TMS and diazepam).

**Figure 3:**
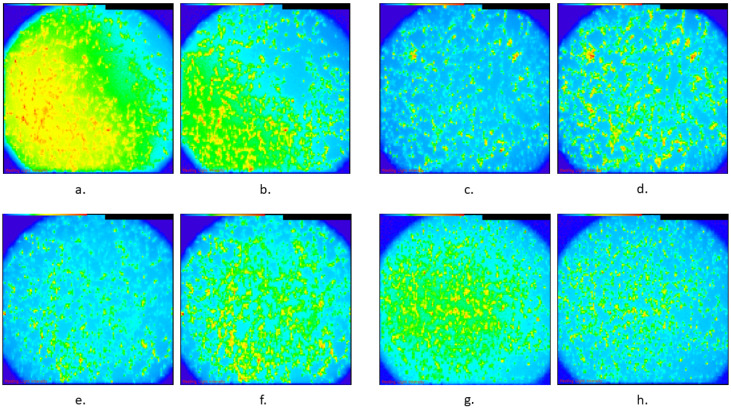
Representative images of intracellular Ca^2+^ in different groups of SH-SY5Y cultures using Fluo-4 AM, showing fluorescence intensity, with blue representing low Ca^2+^ levels and red indicating high Ca^2+^ levels: (**a**). Control group before KCl was added, (**b**). control group after KCl was added, (**c**). rTMS group before KCl was added, (**d**). rTMS group after KCl was added, (**e**). diazepam group before KCl was added, (**f**). diazepam group after KCl was added, (**g**). combination of rTMS and diazepam before KCl was added, and (**h**). combination of rTMS and diazepam after KCL was added.

The distribution characteristics of the %ΔF/F for the four different treatments are shown in Figure 2. The result of the Kruskal–Wallis test was that, overall, the differences in the distributions of ΔF/F % among these four categories were statistically significant (*p*-value < 0.001). When the pairwise differences were assessed in the post hoc analysis after adjusting for multiple comparisons, it was found that the control group, which exhibited the highest median value (24.80; see Table 1A), differed statistically significantly compared with all the remaining three groups (TMS, diazepam, and TMS and diazepam) with adjusted *p*-values of 0.003, 0.002, and <0.001, respectively (Table 1B). Additionally, the TMS group, which exhibited a median value of 16.96 (Table 1A), differed statistically significantly compared with the TMS-and-diazepam group (adjusted *p*-value: 0.013) (Table 1B). A similar result was observed for diazepam vs. TMS and diazepam (adjusted *p*-value: 0.022) (Table 1B). Intracellular Ca^2+^ images are shown in Figure 3, representing every group before and after adding KCl with regard to Ca^2+^ levels using the Ca^2+^-sensitive fluorescent indicator Fluo-4, AM.

## 4. Discussion

In our study, adding KCl resulted in a significant increase in intracellular Ca^2+^ levels, a finding consistent with previous reports [16,20,31]. The group receiving both diazepam (14 μM) and repetitive transcranial magnetic stimulation (rTMS) at 1 Hz showed the lowest change, followed by intermediate fluorescence changes, which were observed in the diazepam group and rTMS groups. Significant differences were found between the TMS group and the TMS-and-diazepam group and between the diazepam group and the TMS-and-diazepam group. These findings suggest that both diazepam and rTMS, particularly when combined, reduce KCl-induced Ca^2+^ influx in SH-SY5Y cultures, highlighting their potentially beneficial effects on seizure-like activity.

Our results were consistent in every experiment, showing a reduction in fluorescence intensity in every category compared to the corresponding control cell cultures, with the lowest values being observed when diazepam was used simultaneously with magnetic stimulation.

Diazepam acts on GABA_A_ receptors as a positive allosteric modulator, increasing chloride ion conductance, hyperpolarizing neurons, and reducing excitability. It has anxiolytic and myorelaxant effects as well as sedative, amnestic, and anticonvulsant properties [32,33]. The SHSY5Y cell line used in our experiments expresses several endogenous sodium voltage-gated channels, GABA_A_ receptors, as well as the ionotropic receptors N-methyl-D-aspartate (NMDA) and α-amino-3-hydroxy-5-methyl-4-isoxazolepropionic acid receptor (AMPA), which are affected by magnetic stimulation [32,34,35,36,37,38,39].

Evidence indicated that diazepam exhibited dose-dependent neuroprotective effects against glutamate-induced neurotoxicity in cortical cultures, with low doses yielding moderate efficacy [8]. When diazepam is administered in conjunction with allopregnanolone, it effectively reverses Ca^2+^ dysregulation caused by tetramethylene disulfotetramine in TETS-intoxicated mice and hippocampal neurons; in contrast, diazepam alone was only effective at high concentrations [25].

In one in vitro experiment, researchers used Fluo-4 to measure changes in Ca^2+^ oscillations in primary neurons after implementing epileptiform activity using either low magnesium or 4-aminopyridine. The researchers tested several antiepileptic drugs and found that diazepam at a concentration of 30 μΜ repressed the development of Ca^2+^ oscillations [40]. The results align with our findings, where diazepam reduced the levels of intracellular Ca^2+^ after KCl exposure, with a ΔF/F% value of 11.46 compared with 24.80 for the control group.

Repetitive TMS may strengthen or decrease synaptic plasticity, with long-term potentiation (LTP) or long-term depression (LTD) of synaptic strength, respectively. High-frequency TMS, at 5 Hz or more, is excitatory to the human brain, making neurons more likely to fire, while low-frequency TMS, at 1 Hz or less, is inhibitory, making neurons less likely to fire an action potential [41]. Low-frequency TMS has been used to repress seizures in patients with extratemporal lobe epilepsy, with promising results [42]. A study in healthy volunteers found that low-frequency TMS, at 0.9 Hz for 15 min, led to motor cortex depression, a finding with notable significance in epilepsy [43]. Those findings also resemble our results, where cells affected by low-frequency rTMS exhibited a lower rise in intracellular Ca^2+^ after KCl depolarization (%ΔF/F: 16.96). Our post hoc analysis did not show any statistical significance in the %ΔF/F median differences between the diazepam and rTMS groups.

Several in vitro studies have applied magnetic stimulation to the SHSY5Y cell line. In one study, the researchers examined different parameters of rTMS, namely, the frequency and duration, when used on differentiated human neuroblastoma cells. They concluded that the intracellular reactive oxygen species levels decreased as the rTMS duration increased [44]. Another study showed that rTMS had an impact on tyrosine hydroxylase activity and the amount of dopamine, L-DOPA, and noradrenaline, with either an increase or a decrease, depending on the frequency and duration of the session [45]. Moreover, a study on SHSY5Y cells, differentiated with retinoic acid, showed that rTMS at a high frequency of 5 Hz activated the cAMP-CREB pathway, leading to higher levels of cAMP and elevated phosphorylation of the cellular transcription factor. The researchers also used ketamine, a channel blocker of the NMDA receptors, and lithium to elucidate any interactions between the drugs and magnetic stimulation. According to their findings, lithium decreased the cellular response to rTMS, while ketamine displayed the opposite results [46].

A study found that low-frequency rTMS reduced the incidence and frequency of seizures in pilocarpine-induced status epilepticus rat models via the AMPAR GluA1–STIM–Ca^2+^ pathway, while it may also have neuroprotective effects, reducing apoptosis and cell damage in hippocampal neurons, where an epilepsy model was established using a Mg^2+^-free bathing solution [47]. A brief review examining the use of rTMS and pharmacotherapy in vitro showed very few eligible studies [13]. To our knowledge, there are no in vitro studies investigating the combination of rTMS with commercially approved antiepileptic drugs. Only one in vitro study has implemented rTMS in combination with a GABA_B_ receptor antagonist in an epilepsy model. The researchers used rodent brain slices and performed low-frequency rTMS (1 Hz) in addition to the GABA_B_ receptor antagonist CGP 55845 and concluded an increased latency of ictal-like epileptiform discharges, which was most pronounced when low-frequency stimulation was used alongside the GABA_B_ receptor antagonist [48]. In our research, we studied the combined effects of rTMS and diazepam and also concluded that the combination of rTMS and pharmacotherapy showcased the most prominent results, with the lowest rise in intracellular Ca^2+^ after the provoked excitation by KCl compared with the control cell cultures, with a %ΔF/F value of −0.44 compared with 24.80 for the control group. Our post hoc analysis between the %ΔF/F values of the TMS and diazepam group and the groups of TMS and diazepam individually also exhibited adjusted *p*-values of 0.013 and 0.022, respectively (Table 1).

Our study had certain limitations. While we made a great effort in keeping all conditions identical throughout the experiments, small fluctuations in environmental temperature may have exerted any variations. Our experiments were conducted using a drug concentration selected for its ability to produce robust responses in controlled in vitro settings, rather than mirroring therapeutic dosing levels. Future in vivo studies should determine clinically relevant or minimally effective dosing parameters to support clinical translation. Although we utilized human-derived mature neurons, in vitro neuronal cultures do not replicate the three-dimensional structure, synaptic connections, or interactions, between diverse cell types, such as astrocytes or microglia that are present in the human brain, which may affect the physiological accuracy of Ca^2+^ data recordings, limiting its direct applicability to in vivo seizure activity. Future research should employ mixed cultures or organoid models with a three-dimensional architecture to more closely mimic the human brain’s structure and function, evaluating their impact on treatment outcomes. The diversity of cell types may play a critical role in regulating brain activity, including neurotransmitter dynamics and Ca^2+^ signaling, which could significantly affect the outcomes of rTMS and diazepam or any other relevant pharmacological interventions.

## 5. Conclusions

The combination of diazepam and low-frequency repetitive transcranial magnetic stimulation (rTMS) resulted in a more pronounced decrease in intracellular Ca^2+^ levels than with either treatment alone, suggesting that their synergistic effect may prove beneficial in the management of epilepsy. These findings underline the value of further preclinical and clinical research into these compounds’ combined therapeutic potential in epilepsy treatment, especially for patients with drug-resistant epilepsy.

## Figures and Tables

**Table 1:** (**A**) Descriptive statistics are displayed regarding the fluorescence intensity of Ca^2+^ influx for the four treatment categories (control, TMS, diazepam, and TMS and diazepam). (**B**) Post hoc pairwise comparisons are displayed based on Dunn’s test. Specifically, the values of Dunn’s correction are provided along with the respective unadjusted *p*-values and their corresponding adjusted *p*-values using the Benjamini–Hochberg procedure to account for multiple comparisons.

**(A)**				
**Group**		**Median**	**Mean**	**Std. Deviation**
Control		24.80	30.98	20.37
TMS		16.96	13.55	15.55
Diazepam		11.46	13.35	17.14
TMS + diazepam		−0.44	3.70	12.48
**(B)**				
**Group 1 vs. Group 2**		**Dunn’s Test Statistic**	**Unadjusted** ***p*-Value**	**Adjusted** ***p*-Value**
Control	TMS	28.433	0.002	0.003
	Diazepam	30.800	0.001	0.002
	TMS + diazepam	51.967	<0.001	<0.001
TMS	Diazepam	2.367	0.792	0.792
	TMS + diazepam	23.533	0.009	0.013
Diazepam	TMS + diazepam	21.167	0.018	0.022

## Data Availability

All the data supporting the findings of this study are available from the corresponding author upon reasonable request.

## Abbreviations

The following abbreviations are used in this manuscript:

rTMS	Repetitive transcranial magnetic stimulation
DRE	Drug-resistant epilepsy
GABA	Gamma-aminobutyric acid
MS	Multiple sclerosis
SLEs	Seizure-like events
EMEM	Eagle’s Minimum Essential Medium
FBS	Fetal bovine serum
DMEM	Dulbecco’s Modified Eagle’s Medium
DMSO	Dimethylsulfoxide
BDNF	Brain-derived neurotrophic factor
NB	Neurobasal medium
LTP	Long-term potentiation
LTD	Long-term depression
NMDA	N-methyl-D-aspartate
AMPA	α-amino-3-hydroxy-5-methyl-4-isoxazolepropionic acid
cAMP	Cyclic adenosine monophosphate
